# Site-oriented conjugation of poly(2-methacryloyloxyethyl phosphorylcholine) for enhanced brain delivery of antibody

**DOI:** 10.3389/fcell.2023.1214118

**Published:** 2023-10-18

**Authors:** Jie Ren, Chloe E. Jepson, Sarah L. Nealy, Charles J. Kuhlmann, Satoru Osuka, Stella Uloma Azolibe, Madison T. Blucas, Yoshiko Nagaoka-Kamata, Eugenia Kharlampieva, Masakazu Kamata

**Affiliations:** ^1^ Department of Microbiology, University of Alabama at Birmingham, Birmingham, AL, United States; ^2^ Department of Chemistry, University of Alabama at Birmingham, Birmingham, AL, United States; ^3^ Department of Neurosurgery, University of Alabama at Birmingham, Birmingham, AL, United States; ^4^ Department of Pathology, School of Medicine, University of Alabama at Birmingham, Birmingham, AL, United States

**Keywords:** antibody, brain disease, trastuzumab, biofunctionality, poly 2-methacryloyloxyethyl phosphorylcholine, site-oriented conjugation, brain delivery

## Abstract

Antibody therapeutics are limited in treating brain diseases due to poor blood-brain barrier (BBB) penetration. We have discovered that poly 2-methacryloyloxyethyl phosphorylcholine (PMPC), a biocompatible polymer, effectively facilitates BBB penetration via receptor-mediated transcytosis and have developed a PMPC-shell-based platform for brain delivery of therapeutic antibodies, termed nanocapsule. Yet, the platform results in functional loss of antibodies due to epitope masking by the PMPC polymer network, which necessitates the incorporation of a targeting moiety and degradable crosslinker to enable on-site antibody release. In this study, we developed a novel platform based on site-oriented conjugation of PMPC to the antibody, allowing it to maintain key functionalities of the original antibody. With an optimized PMPC chain length, the PMPC-antibody conjugate exhibited enhanced brain delivery while retaining epitope recognition, cellular internalization, and antibody-dependent cellular phagocytic activity. This simple formula incorporates only the antibody and PMPC without requiring additional components, thereby addressing the issues of the nanocapsule platform and paving the way for PMPC-based brain delivery strategies for antibodies.

## Introduction

Antibodies have been widely employed as therapeutics and diagnostics due to their high binding affinity, specificity, and biocompatibility compared with other molecules (Lu et al., 2020). To date, more than 100 antibody-based therapeutics have been approved by the Food and Drug Administration (FDA) for treatments of various diseases, including cancers (Zahavi and Weiner, 2020), autoimmune diseases (Yasunaga, 2020), infectious diseases (Hurt and Wheatley, 2021), and metabolic diseases (Ahrens, 2011). However, the merit of antibody therapeutics is negated in treating brain diseases, even though many antibodies have been proven to target the pathological antigens of brain diseases effectively *ex vivo* (Sehlin et al., 2019; Henderson et al., 2020; Perneczky et al., 2023). Such a limitation mainly results from the blood-brain barrier (BBB) that separates the peripheral circulating system from the brain via tight junctions and restricted transcytosis (Kadry et al., 2020; Ruano-Salguero and Lee, 2020). Generally, only 0.1%–0.2% of antibodies infused to the blood can penetrate the BBB and be deposited in the brain (Kouhi et al., 2021). Therefore, an effective and biocompatible brain delivery system would boost antibody applications and change the landscape of treatment regimens for brain diseases.

The current strategies mainly utilize receptor-mediated transcytosis through the endothelial cells in the BBB (Pulgar, 2019). BBB-penetrating ligands have been developed to target the receptors on the BBB, including glucose transporter-1 (Min et al., 2020), low-density lipoprotein receptor-related protein-1 (Gao et al., 2022), and transferrin receptor (Choudhury et al., 2018). Antibodies are fused or conjugated with these ligands to facilitate their entry into the brain (Boado, 2022). Yet the ligands are either derived from microbes/toxins, such as rabies virus (Su et al., 2020), that are highly immunogenic or endogenous proteins like lipoproteins (Zhu et al., 2022) that are highly hydrophobic or charged. Thus, antibodies with those ligands suffer from undesirable changes in surface properties, which may compromise antibody blood circulation and functions and expose the patients to high risks.

Poly 2-methacryloyloxyethyl phosphorylcholine (PMPC) is a biocompatible, non-immunogenic polymer approved by the FDA as a coating material in transplantable devices (Fujiwara et al., 2020). Our group has discovered that PMPC polymers can interact with nicotinic acetylcholine receptors and choline transporters similarly to acetylcholine and choline (Wu et al., 2019). Such interactions facilitate receptor-mediated transcytosis by endothelial cells of the BBB, enhancing PMPC delivery to the brain. Utilizing PMPC and designated crosslinkers, we have encapsulated various macromolecular cargos within PMPC shells through *in situ* polymerization, termed MPC nanocapsules, and demonstrated prolonged blood circulation, reduced immunogenicity, and enhanced brain delivery in mice and non-human primates (Han et al., 2019; Wen et al., 2019; Xu et al., 2019; Zou et al., 2020). Moreover, the nanocapsule surface can be modified with target-specific ligands, which further guide it to disease sites after brain entry. For instance, we encapsulated the therapeutic antibody rituximab (RTX) that targets CD20 of B-cell lymphoma into MPC nanocapsules with degradable crosslinkers, followed by conjugation of CXCL13 as a ligand (nRTX^CXCL13^) on the surface to target B-cell lymphomas (Wen et al., 2019). We demonstrated superior brain delivery via MPC nanoencapsulation, tumor targeting through CXCL13, and complete elimination of brain-metastasized B-cell lymphoma by releasing RTX in the brain. This could not be achieved by native RTX, suggesting that PMPC engineering is a powerful strategy for brain delivery of therapeutic antibodies.

The current method of MPC nanoencapsulation fabricates an MPC network surrounding the surface of antibodies to form an MPC shell (Yan et al., 2010). The polymer network protects the antibody from immune surveillance and minimizes on-target/off-tumor toxicity but also conceals the epitope recognition and biological activities, necessitating the addition of targeting ligands, such as CXCL13, and on-site antibody release following the destruction of the shell via degradable crosslinkers. However, the disease-associated microenvironment often lacks or differs in stimuli, such as acidity and overexpression of certain enzymes that can trigger the degradation of crosslinkers (Ugrumov, 2020). Moreover, some types of cancer, like glioblastoma, differ in the microenvironment, leading to difficulty in selecting targeting ligands (Pandey et al., 2022). In those cases, it is more favorable to endow antibodies with enhanced BBB penetrability while maintaining their biofunctionality. Inspired by ligand antibody fusion methodology, we hypothesize that direct conjugation of PMPC to antibodies can preserve epitope recognition while enhancing brain entry.

**FIGURE 1:**
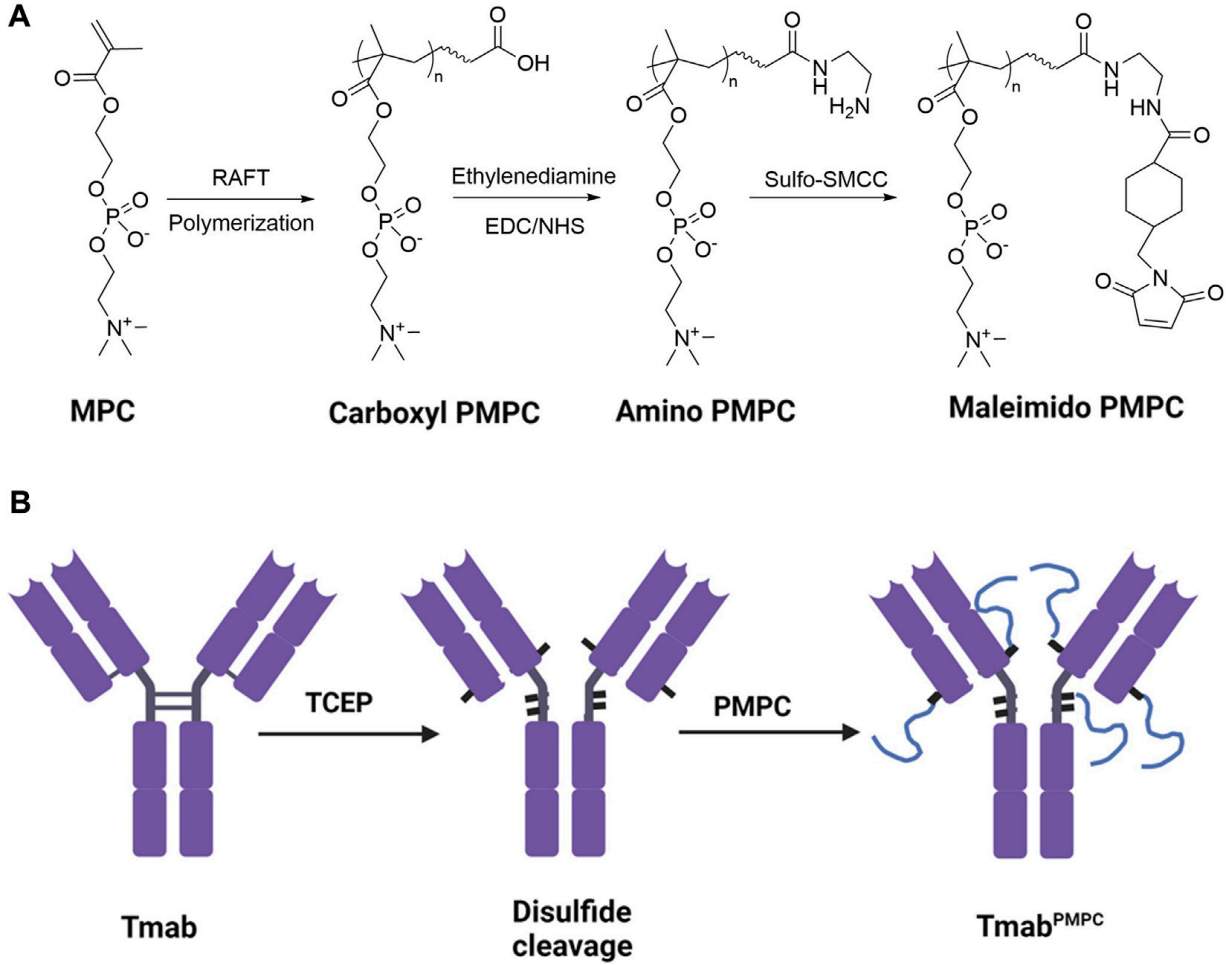
Synthetic process of PMPC polymers and Tmab^PMPC^s. **(A)** Synthetic process of thiol-reactive maleimide-modified PMPC. MPC was first polymerized following a RAFT polymerization to obtain Carboxyl-terminated PMPC. Then the end group was converted to an amino group with Ethylenediamine modification, followed by converting the amino group to maleimide group by Sulfo-SMCC to obtain maleimide-modified PMPC. **(B)** Synthetic process of Tmab^PMPC^ production. The interchain disulfide bonds of Tmab were first cleaved by TCEP to create thiol groups, followed by reaction with maleimide-modified PMPC to obtain Tmab^PMPC^.

The majority of FDA-approved therapeutic antibodies are IgG1-based (Tang et al., 2021). In the IgG1 structure, four interchain disulfide bonds are located in the hinge and near-hinge area distant from the functional binding epitopes in the Fab and Fc domains (Hagihara and Saerens, 2014). Those disulfide bonds can be cleaved into thiol groups by reductive reagents without disturbing the integrity or binding affinity of the antibody, which can be further coupled to thiol-reactive species (Walsh et al., 2021). It has been reported that site-specific conjugation of polyethylene glycol to the thiol groups of the Fab does not interfere with epitope recognition (Chapman et al., 1999). We reason that PMPC can be conjugated to the thiol groups cleaved from the interchain disulfide bridges of IgG1 antibodies to avoid masking the binding epitopes. We synthesized a series of PMPC polymers with three lengths by reversible addition-fragmentation chain-transfer (RAFT) polymerization. Then, we conjugated these polymers to trastuzumab (Tmab), which targets human epidermal growth factor receptor 2 (HER2) (Figure 1). We investigated the impact of PMPC conjugation length on target recognition, cellular internalization, antibody-dependent cellular phagocytosis (ADCP), and brain delivery efficiency. With an optimal length of PMPC, we achieved effective brain delivery of conjugated antibody nearly 5 times higher than the native counterpart with its biofunctionality maintained. This simple strategy paves the way for novel approaches to the brain delivery of antibody therapeutics toward clinical practice.

## Materials and methods

### Materials

Unless otherwise noted, all chemicals were purchased from Sigma-Aldrich (St. Louis, MO). Trastuzumab was purchased from Bio X Cell (Lebanon, NH). Human IgG1, Corning transwell with permeable polyester membrane inserts, and Pierce bicinchoninic acid (BCA) colorimetric protein assay kit were purchased from Thermo Fisher Scientific (Waltham, MA). 5-Carboxytetramethylrhodamine (TAMRA) N-hydroxysuccinimide (NHS) Ester and Alexa fluor 647 (AF647) NHS ester were purchased from Click Chemistry Tools (Scottsdale, AZ). PD-10 desalting column, zeba desalting column, dialysis tubing (Molecular weight (MW) cutoff 3 KD), 1-Ethyl-3-[3-dimethylaminopropyl] carbodiimide hydrochloride (EDC), NHS, 2,2′-Azobis [2-(2-imidazolin-2-yl)propane]dihydrochloride (VA-044), Sulfosuccinimidyl-4-(N-maleimidomethyl)cyclohexane-1-carboxylate (Sulfo-SMCC), and Tris (2-carboxyethyl)phosphine hydrochloride (TCEP) were purchased from Fisher scientific. SKOV-3, THP-1, Jurkat, and bEnd.3 cells were purchased from American Type Culture Collection (ATCC). The parental Jurkat cells were transduced with a lentiviral vector encoding human HER2 and mCherry to obtain HER2+ mCherry + Jurkat cells, as reported (Wen et al., 2019). Human glioblastoma cell line, JX14P cells were derived from a glioblastoma biopsy (Osuka et al., 2021). Iscove’s Modified Dulbecco’s Medium (IMDM), GlutaMax, Antibiotic-Antimycotic solution, Trypsin-EDTA, and heat-inactivated fetal bovine serum (FBS) were obtained from Corning (Corning, NY). Cell Counting Kit-8 (CCK-8) was purchased from ApexBio Technology LLC (Houston, TX). Corning transwell with permeable polyester membrane, CellTrace™ Far Red Cell Proliferation Kit, Pierce BCA protein assay kit, Lysotracker deep red, and 96 ELISA well-plates were purchased from Thermo Fisher Scientific. The capture (goat anti-human IgG Fab) and detection (HRP-conjugated goat anti-human IgG (H + L)) antibodies were purchased from BioRad Laboratories (Hercules, CA). NOD.Cg-*Prkdc* ^*scid*^ *Il2rg* *^tm1Wjl^/SzJ* (NSG) mice were purchased from the Jackson Laboratory (Bar Harbor, ME). Aspartate aminotransferase (AST) and alanine aminotransferase (ALT) colorimetric assay kits were purchased from Cayman Chemicals (Ann Arbor, MI). Mouse anti-glial fibrillary acidic protein (GFAP) antibody was purchased from Biolegend (San Diego, CA). Rabbit anti-ionized calcium binding adaptor molecule 1 (Iba1) antibody was purchased from FujiFILM Pure Chemical Corporation (Richmond, VA). AF647 labeled donkey anti-mouse IgG and Alexa fluor 488 (AF488) labeled goat anti-rabbit IgG antibodies were purchased from Jackson Immunoresearch laboratories, Inc. (West Grove, PA).

### Animals

Animal research described in the study was approved by UAB IACUC (APN-21938) and was conducted in accordance with guidelines for the housing and care of laboratory animals of the National Institutes of Health and Association for Assessment and Accreditation of Laboratory Animal Care International. The mice used in this study are female NSG mice between 8–10 weeks. All animals were housed in autoclaved cages with isolators with individual air sources supplemented with irradiated standard rodent chow (LabDiet, 5LJ5) and Hydropac. In total, eighteen mice were used in the biodistribution experiment with three mice per group. Twelve mice were used to evaluate toxicity with three mice per group. The integrity of the BBB was tested by using twelve mice with two mice per group.

### Synthesis of PMPC polymers

PMPC polymers with carboxyl end groups were synthesized following a fast RAFT polymerization procedure (Gody et al., 2015) with MPC as the monomer, 4-Cyano-4-(phenylcarbonothioylthio)pentanoic acid (CPPA) as the chain transfer agent (CTA), and VA-044 as the initiator. Briefly, 2 mM MPC and VA-044 were mixed and dissolved in 500 μL cell culture grate water (Corning), followed by CPPA (10% w/v in dimethyl sulfoxide, (DMSO). The molar ratios of VA-044 to MPC were set to 1,000:1, 500:1, and 250:1, while those of CPPA to MPC were set to 200:1, 100:1, 50:1 to synthesize PMPC200, PMPC100, and PMPC50. The polymerization was allowed to stir at 95°C for 3 min, followed by cooling down in liquid nitrogen to stop the reaction. The pH value of the reaction solution was then adjusted to 12 with 2M NaOH, followed by incubation at room temperature for 2 h to remove the dithiocarbonate group of CTA. Then the solutions were dialyzed against water for 24 h to remove the unreacted materials. The obtained PMPC polymers were stored in an aqueous solution at −80°C for the next step.

### Synthesis of maleimide-modified PMPC polymers

The terminal groups of PMPC polymers were first converted into primary amine groups. The carboxyl groups were activated by EDC and NHS at molar ratios to PMPC of 10:1 and 4:1, respectively, in 2-(N-morpholino) ethanesulfonic acid (MES) buffer (pH 5.0, 100 mM). The activation was allowed to stir at 4°C for 1 h, followed by the addition of ethylenediamine (molar ratio to PMPC at 20:1). The pH value was quickly adjusted to 8.0, followed by stirring at room temperature for 2 h. The reaction mixture was dialyzed against water (MW cutoff: 3 KD) for 24 h to remove the unreacted species. The resultant polymer solutions were reacted with Sulfo-SMCC (molar ratio to PMPC at 2:1) at 4°C for 2 h and then passed through Zeba desalting columns to obtain maleimide-modified PMPC polymers. The polymer solution was lyophilized and stored at −80°C.

### Fourier-transform infrared spectroscopy (FT-IR)

FT-IR analysis of the PMPC samples was carried out using a Bruker Alpha ATR-FTIR spectrometer. The ATR accessory was used for signal enhancement of chemical species by internal total reflectance. The number of scans was set to 32 with a resolution on four for all samples analyzed. All samples were dried in a vacuum oven at room temperature for 24 h prior to performing sample scans.

### Proton nuclear magnetic resonance (^1^H-NMR)

The PMPCs were characterized by nuclear magnetic resonance spectroscopy (^1^H NMR) analysis, which can be used to calculate the average number of repeat units in the PMPC backbone (degree of polymerization). For all PMPC samples, ^1^H NMR spectra were collected using a Bruker 400 MHz NMR spectrometer in proton scan mode. The PMPC samples were prepared in D_2_O (99.8%) at a concentration of 1 mg/mL and analyzed at room temperature with 64 scans. Additionally, the percent conversion from PMPC-COOH to PMPC-Mal was calculated by ^1^H-NMR analysis, and the PMPC-Mal samples were prepared for ^1^H-NMR with 256 number of scans to resolve the end group proton signals appearing at 6.8 ppm.

### Fluorescent labeling of Tmab

Tmab was modified by TAMRA or AF647 to the surface lysine groups, respectively. For TAMRA conjugation, TAMRA NHS ester (1% w/v in DMSO) was added to Tmab (2 mg/mL) solution in PBS at a molar ratio of 10:1 and incubated at 4°C for 2 h. The Tmab solution was then passed through a PD-10 desalting column to remove the unconjugated TAMRA and stored at 4°C. The conjugation of AF647 followed the protocol above with an AF647 NHS ester to Tmab molar ratio of 2:1.

### Conjugation of PMPC polymers to Tmab

The interchain disulfide bridges of Tmab were converted into thiol groups by TCEP following a published protocol (Bahou et al., 2018). 2 mL of Tmab (2 mg/mL) in PBS was added by 0.16 mL of TCEP (1 mg/mL) (molar ratio: 40:1), and 20 µL of EDTA (0.5 M), followed by incubation at 37°C for 2 hours. The Tmab solution was then purified by passing through a PD-10 column to remove extra TCEP. The concentration of Tmab in the solution was determined by BCA assay, as described below. The thiol group number per antibody was measured by the Ellman assay as published (Hoenicka, 1968). Then maleimide-modified PMPC polymers were added to the Tmab solution (molar ratio of PMPC to Tmab at 100:1) and incubated at 4°C for 12 h for PMPC conjugation. The unconjugated polymers were removed by passing through an ultrafilter (MW cutoff: 100 KD) five times. The resultant Tmab^PMPC^s were stored at 4°C for further experiments. The synthesis of fluorescence labeled Tmab^PMPC^s followed the same protocol as described above with AF647 or TAMRA labeled Tmab. To minimize the potential detachment of PMPC through retro-Michael addition, all the PMPC conjugated samples were stored at 4°C and were used for all the *in vivo* and *in vitro* experiments within 24 h post-PMPC conjugation.

### Synthesis of Tmab encapsulated MPC nanocapsule (nTmab)

Tmab was encapsulated into nanocapsules via *in situ* polymerization as reported previously (Wu et al., 2019) using MPC (50% w/v in water) as the monomer, glycerol dimethacrylate (GDMA, 5% w/v in DMSO) as the crosslinker, ammonium persulfate (APS, 10% w/v in PBS) as the initiator, tetramethylethylenediamine (TEMED, 10% w/v in PBS) as the catalyst. Tmab in PBS (1 mg/mL) was mixed with MPC, GDMA, and APS at the molar ratios of 1: 12,000: 1,200: 2.000, after which the polymerization was initiated by adding TEMED solution (molar ratio 1:1 to APS). The mixture was incubated at 4°C for 2 h, followed by dialysis against PBS to remove unreacted reagents. The resultant nTmab was stored at 4°C for further experiments.

### Protein concentration assay

Antibody content in the form of Tmab^PMPC^ and nTmab was determined by BCA colorimetric protein assay. Briefly, a tartrate buffer (pH 11.25) containing BCA (25 mM), CuSO_4_ (3.2 mM), and Tmab samples were incubated at 37°C for 2 h. After the reaction was cooled to room temperature, the absorbance reading at 562 nm was determined with a UV/Vis spectrometer. Native Tmab was used to generate a standard curve to calculate the Tmab concentration in samples.

### SDS-PAGE and dynamic light scattering (DLS) measurement

The conjugation of PMPC was validated through SDS-PAGE gel electrophoresis (4%–12% Precast gel, Nakalai United States, San Diego, CA). 5 μg of Tmab, Tmab after disulfide-cleavage, or Tmab^PMPC^s were mixed with 5x protein loading dye. Gel electrophoresis was run in 1x SDS running buffer (Nakalai United States) at 160 mV for 2 hours. Gels were stained overnight in Coomassie blue staining solution, followed by destaining in a methanol/acetic acid mixture to visualize the bands. Gel images were taken with an iBright 1,500 gel imaging system (Thermo Fisher Scientific, Waltham, MA). The size distributions of Tmab^PMPC^s and nTmab were determined by DLS at the Tmab concentration of 1 mg/mL in PBS solutions at 25°C with a DynaPro NanoStar II (Wyatt, Santa Barbara, CA). DLS measurements were performed three times to determine the average diameter.

### Cell culture and cellular viability measurement

THP-1 and bEnd.3 cells were cultured in IMDM supplemented with 10% FBS, 1% GlutaMax, and 1% Antibiotic-Antimycotic. JX14P cells were cultured in Dulbecco’s Modified Eagle Medium Nutrient Mixture F-12 (Nassour et al., 2022). HER2+ mCherry + Jurkat cells were cultured in IMDM supplemented with 10% FBS, 1% GlutaMax, 1% Antibiotic-Antimycotic, Doxycycline (1 μg/mL), and neomycin (250 μg/mL).

To measure the cytotoxicity of Tmab^PMPC^, bEnd.3 cells and JX14P cells were selected as the model BBB endothelial cells and glial cells, respectively. bEnd.3 cells and JX14P cells were seeded to 96-well plates (10,000 cells per well). After 24 h, 100 μL PMPC200 or Tmab^PMPC200^ in cell culture medium (concentration ranging from 5 to 1,000 μg/mL) was added to each well and incubated at 37°C for 72 h. The cell viability was then measured with Cell Counting Kit-8 (UV adsorption at 450 nm).

### Binding affinity assay

To investigate the binding affinity of Tmab^PMPC^s to HER2, SKOV-3 was used as the HER2+ cell line. SKOV-3 cells were seeded on glass coverslips. After 24 h, cells were incubated with AF647 labeled Tmab^PMPC^s, Tmab, or nTmab at the final Tmab concentration of 1 μg/mL at 37°C for 2 h, followed by 4% paraformaldehyde for 15 min at room temperature for fixation after three washes with PBS. Nuclei were stained by 10 μg/mL Hoechst 33342 at room temperature for 20 min. Fluorescent images were taken with a Nikon A1R/SIM confocal microscope (Nikon, Melville, NY).

The dissociation constant (Kd) of each Tmab sample was determined as follows: 2 × 10^5^ SKOV-3 cells in FACS buffer (5% FBS in PBS, 2 mM EDTA, 0.02% NaN_3_) were incubated with AF647 labeled Tmab, Tmab^PMPC^s, and nTmab with a Tmab concentration gradient ranging from 0.002 to 400 nM at 4°C for 1 h. The cells were then washed and fixed. The mean fluorescent intensity (MFI) of AF647 was measured with a FACSymphony flow cytometer (BD Biosciences, San Jose, CA). The Kd was determined by analyzing the profiles curved from the MFI at each concentration.

### ADCP assay

The ADCP ability of Tmab^PMPC^ was determined as follows: THP-1 cells were used as the phagocytic cell line, while HER2+ mCherry + Jurkat cells were used as the target cell line. THP-1 cells were labeled with CellTrace™ Far Red Cell Proliferation Kit at room temperature for 30 min 5 × 10^5^ Jurkat cells were incubated with Tmab^PMPC^s, Tmab, or nTmab at room temperature for 30 min and then added to Far-red labeled THP-1 cells (1 × 10^5^). The cells were incubated at 37°C for 2 hours and then fixed with 4% formaldehyde in PBS. The phagocytic levels were determined by flow cytometry.

### Tmab internalization

The levels of Tmab internalization were determined in SKOV-3 cells and bEnd.3 cells. For the assay in SKOV-3 cells, TAMRA-labeled Tmab^PMPC^s, Tmab, or nTmab (50 μg/mL each) were incubated with SKOV-3 cells at 37°C for 24 h in a 12-well plate, followed by three PBS washes. The cells were then incubated with AF488-labeled anti-human IgG antibody (10 μg/mL) at 4°C for 30 min to stain the Tmab bound on the surface of cells. Following fixation by 4% formaldehyde, the nuclei were stained by Hoechst 33342. The fluorescent images were taken with a Nikon A1R/SIM confocal microscope. Pearson correlation coefficients were obtained by analyzing three different areas with the colocalization-finder plugin in ImageJ.

For the assay in bEnd.3 cells, cells were incubated with TAMRA-labeled Tmab^PMPC^s, Tmab, or nTmab (50 μg/mL each) at 37°C for 2 h, followed by three PBS washes. The cells were then harvested by trypsin-EDTA digestion and fixed with 4% formaldehyde in PBS solution. The MFI was measured with a FACSymphony flow cytometer as above.

### Intracellular trafficking

The intracellular trafficking of Tmab^PMPC^s was investigated by measuring the co-localization of internalized Tmab and the late endosome/lysosome. bEnd.3 cells were seeded onto glass coverslips, followed by adding TAMRA-labeled Tmab samples to each well at the final Tmab concentration of 50 μg/mL. The cells were incubated at 37°C for 4 h, followed by three PBS washes. Lysotracker deep red in the fresh medium was added to each well at the final concentration of 50 nM and incubated at 37°C for 30 min. The cells were then washed with PBS and fixed. Nuclei were stained with Hoechst 33342. The fluorescent images of the cells were taken with a Nikon A1R/SIM confocal microscope. Pearson correlation coefficients were obtained by analyzing three different areas with the colocalization-finder plugin in ImageJ.

### Transwell assay

The transwell assay to determine the levels of transcytosis was performed as follows: bEnd.3 cells were seeded on the polyester membrane inserts (pore size, 0.4 μm) of transwells at 10^5^ cells/well. The cell culture media in the apical and basolateral compartments were replaced every 3 days for 3 weeks. TAMRA-labeled Tmab samples in 150 μL of culture medium were added to each insert of transwells at Tmab concentration of 0.05 mg/mL while the medium volume in the basolateral compartment was set to 600 μL. 200 μL of the medium in the basolateral chamber was collected at 0, 2, 4, 6, 8, and 10 h after adding samples and replenished with 200 μL fresh medium. The fluorescent intensity of the medium collected from the basolateral compartment was analyzed by a Varioskan LUX plate reader. The profile of the penetration rate as a function of time was plotted. For the transwell assay with PMPC competition, PMPC200 and Tmab^PMPC200^ in cell culture medium were mixed at the PMPC molar ratio of 20:1 or 200:1 and then added to the apical chamber. The profile of the penetration efficiency (%) vs. time was plotted and fitted with the equation:

$$PEn\;\left(\%\right)=\frac{\text{FLb}\times 600}{\text{FLa}\times 150}\times 100\%+\frac{PE\left(n-1\right)\left(\%\right)\times 200}{600}$$

Where PE*n* (%) represents the penetration efficiency at a certain time point; FLb represents the fluorescent intensity of the medium taken from the basolateral chamber at that time point; PE(*n*-1) (%) represents the penetration efficiency (%) at the previous time point; PE0 (%) equals to zero.

To validate the epitope recognition ability of Tmab after BBB penetration, the medium in the basolateral chamber was collected 10 hours post the sample addition, diluted ten times with FACS buffer, and then incubated with SKOV-3 cells (2 × 10^5^) in FACS buffer at 4°C for 1 h. The MFI was measured by FACSymphony flow cytometer as below; briefly, 2 × 10^5^ SKOV-3 cells in FACS buffer (5% FBS in PBS, 2 mM EDTA, 0.02% NaN_3_) were incubated with AFTmab, Tmab, or PBS with a Tmab concentration gradient ranging from 0.002 to 400 nM at 4°C for 1 hour. The cells were then washed with FACS buffer three times, followed by incubation with AF488 labeled anti-human IgG antibody (1 μM) at 4°C for 2 hours. After washing with FACS buffer three times, the cells were fixed in fixation buffer containing 4% paraformaldehyde. The MFI of AF488 was measured with a FACSymphony flow cytometer. The Kd was determined by analyzing the profiles curved from the MFI at each concentration. The Kd values of AFTmab and Tmab were 0.71 nM and 0.60 nM, respectively, indicating that AF647 labeling did not disrupt the binding affinity of Tmab.

### Biodistribution

The biodistribution was investigated by optical imaging. NSG mice were intravenously administered via the retro-orbital vein with 100 μL of AF647 labeled Tmab, Tmab^PMPC^s, or nTmab (10 mg/kg). *In vivo* fluorescent imaging was performed with an IVIS Lumina II (Perkin Elmer, Waltham, MA) 24 h post-injection (Ex. = 640 nm, Em. = 710 nm). Organ imaging of the heart, liver, spleen, lungs, kidneys, and brain was performed following PBS perfusion. Briefly, mice were euthanized via isoflurane overdose (>5% v/v in air) as approved by UAB IACUC. The mice were cut open below the diaphragm, and the rib cage was cut rostrally on the lateral edges to expose the heart. A small hole was cut in the left ventricle, and the needle was inserted into the aorta and clamped, and then the right atrium was cut to allow flow. The animal was transcardially perfused with PBS wash for 5 min (approx. 20 mL) until the liver (pale yellow), kidney (pale yellow), lung (white), and spleen (pink) were cleared of blood. The quantitative measurement of the fluorescence in average radiant efficiency (brains) or total radiant efficiency (other organs) was measured by the region of interest (ROI) tool in Living Image^®^ software.

### Enzyme-linked immunosorbent assay (ELISA)

The delivery of Tmab to the brain was quantified by ELISA. Briefly, NSG mice were intravenously administrated via the retro-orbital vein with 100 μL Tmab, Tmab^PMPC^s, or nTmab (10 mg/kg). The brains were harvested 24 h post-administration following PBS perfusion, homogenized, and centrifuged to collect the brain extract. A 96 well ELISA-plate was coated with anti-human IgG antibody (1 μg/mL in carbonate buffer, pH 9.5) at 4°C overnight, followed by three washes with PBST (0.1% Tween in PBS). Non-specific binding was blocked with blocking buffer (2% BSA in PBST) at room temperature for 2 h, followed by three washes with PBST. The brain extract was then diluted 1,000-fold with blocking buffer and incubated in the well at room temperature for 2 hours, followed by five washes with PBST. Tmab and Tmab^PMPC^s in blocking buffer (concentration ranging from 0 to 20 ng/mL) were used as a standard. HRP-coupled anti-human IgG antibody (10 μg/mL) was added to each well and incubated at room temperature for 1 h, followed by five washes with PBST. Finally, 3,3′,5,5′-Tetramethylbenzidine and H_2_O_2_ solutions were added and incubated at room temperature for 15 min. The UV adsorption at 450 nm was measured with a Varioskan LUX plate reader.

### Evaluation of organ damages

Liver toxicity was determined by measuring the levels of AST and ALT in plasma samples. Mice were intravenously administered with 150 μL of Tmab, PMPC200, or Tmab^PMPC200^ at Tmab dosage of 50 mg/kg or PMPC dosage of 90 mg/kg via the retro-orbital vein. PMPC dosage in PMPC200 is the same as that in Tmab^PMPC200^, as calculated by the molecular weight, average conjugation number of PMPC200 per Tmab, and the molecular weight of Tmab. PBS was injected as a negative control. The blood was collected from the retro-orbital vein either before or 72 h post-treatment into heparin-coated tubes and centrifuged to obtain the plasma as the supernatant. The levels of AST and ALT in plasma samples were measured with Cayman AST and ALT colorimetric activity assay kits.

The levels of Iba1 and GFAP were used to determine neurotoxicity in the brain. The brains were fixed in 4% paraformaldehyde overnight, followed by incubation with 15% and 30% sucrose in PBS for 6 h, respectively. The brains were processed into sections using a vibratome (VT1000S, Leica Biosystems, Dear Park, IL) in PBS with a thickness of 100 μm. Anti-Iba1 and anti-GFAP antibodies were used as primary antibodies. The sections were permeabilized with 1% Triton X-100 in PBS for 5 minutes and blocked with blocking buffer (1% BSA, 0.2% Triton X-100 in PBS) at room temperature for 2 h. The sections were incubated with anti-GFAP (1:200) and anti-Iba1 (1:500) overnight in a blocking buffer at 4°C. Following a wash with 0.2% Triton X-100 in PBS, the sections were incubated with secondary antibodies (AF488 labeled goat anti-rabbit IgG for anti-GFAP and AF647 labeled donkey anti-mouse IgG for anti-Iba1) in blocking buffer for 1 h at room temperature. After washing with 0.2% Triton X-100 in PBS, the sections were analyzed on A1R/SIM confocal microscope. Area fraction quantifications for GFAP and Iba1 staining were measured with ImageJ.

## Results

### Site-oriented conjugation of PMPC to Tmab retains Tmab functionalities

**FIGURE 2:**
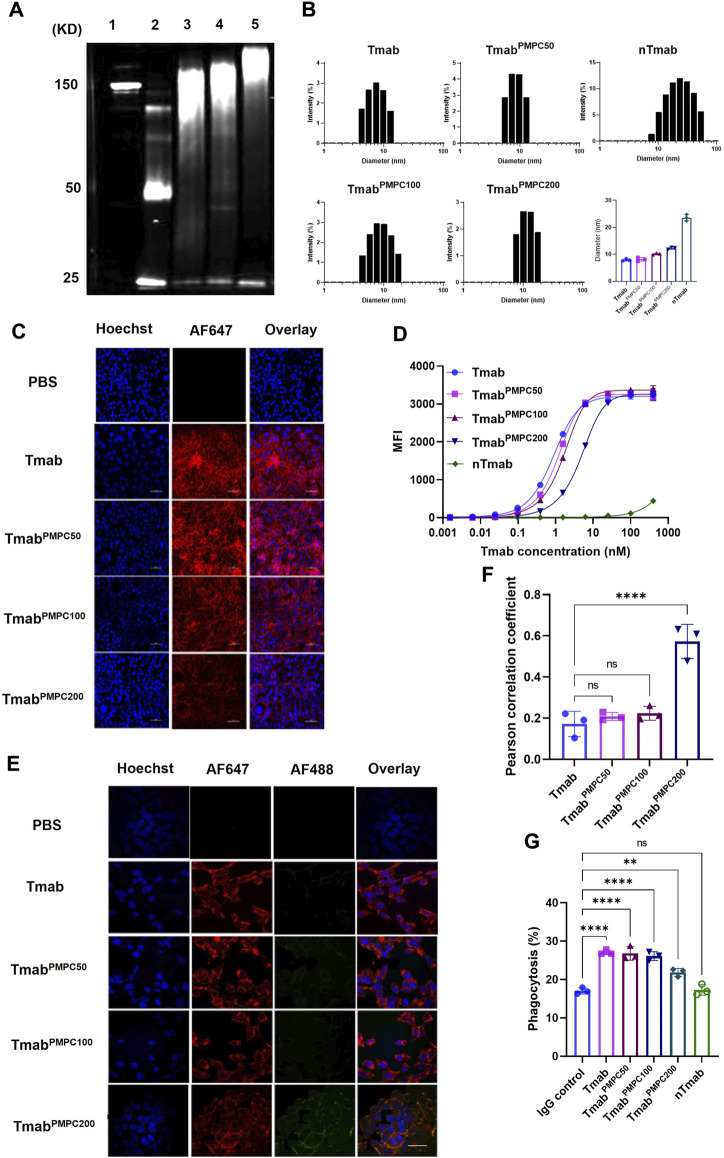
Structural and biofunctional characterizations of Tmab^PMPC^. **(A)** SDS-PAGE images of Tmab, TCEP-reduced Tmab, Tmab^PMPC50^, Tmab^PMPC100^, and Tmab^PMPC100^ (lanes 1–5). **(B)** Size distributions and the average diameters of Tmab, Tmab^PMPC50^, Tmab^PMPC100^, Tmab^PMPC200^, and nTmab determined by DLS measurement in PBS at 25°C. **(C)** CLSM images of AF647 labeled Tmab, Tmab^PMPC50^, Tmab^PMPC100^, and Tmab^PMPC200^ following incubation with SKOV-3 cells. The cell nuclei were labeled by Hoechst 33342 (blue) after 2 hours incubation with each sample at 37°C. The scale bar represents 50 μm. **(D)** Saturation binding curves of Tmab, Tmab^PMPC50^, Tmab^PMPC100^, Tmab^PMPC100^, and nTmab in SKOV-3 cells. **(E)** CLSM images of AF647 labeled Tmab, Tmab^PMPC50^, Tmab^PMPC100^, and Tmab^PMPC200^ binding to SKOV-3 cells. The cell nuclei were stained by Hoechst 33342 (blue) while surface bound Tmab was stained by AF488 labeled anti-human IgG antibody after 24 h incubation with each sample at 37°C. The scale bar represents 50 μm. **(F)** Pearson correlation coefficient of AF488 and AF647 signals in **(E)** figures. Three different areas of each sample were measured. **(G)** Phagocytic percentage of THP-1 cells incubated with HER2+ Jurkat cells pre-treated with Tmab samples for 2 hours. Error bars represent the s.e.m. of triplicate samples (n = 3), ****p < 0.0001. ns: not significant.

As illustrated in Figure 1A, we synthesized PMPC polymers with three different chain lengths via RAFT polymerization. The feed ratio of the monomer MPC to the chain transfer agent was set to 50:1, 100:1, and 200:1, respectively, denoted as PMPC50-COOH, PMPC100-COOH, and PMPC200-COOH. The FT-IR spectra of PMPC-COOH, deprotected PMPC-COOH, and PMPC-Mal showed the bonds in PMPC backbone including C-H, C=O, O–H, N–H, C–N, P=O, P-O-C, P-O, C–O–C, and P–O (Supplementary Figure S1). The degrees of polymerization (DP) of PMPC polymers were calculated by the peak integration ratio of methyl protons (3H, peak a) in PMPC backbone between 0.82–1.15 ppm and phenyl protons (5H, peak m, l, k) of CTA between 7.45–8.02 ppm (Supplementary Figure S1A), which were determined as 37, 58, and 156. The CTA end group was deprotected through hydrolysis to cleave the dithionate group and subsequent Michael addition by MPC monomer, as demonstrated by the disappearance of phenyl proton peaks in ^1^H NMR spectrum (Supplementary Figure S2B). The end group of PMPC polymers was modified with Sulfo-SMCC to produce maleimide-modified PMPC polymers that are thiol-reactive, denoted as PMPC50-Mal, PMPC100-Mal, and PMPC200-Mal (Supplementary Figure S2C). The modification ratios of maleimide groups were determined as 60.9, 51.9, 11.3 by the peak integration ratio of methyl protons (3H, peak a) in PMPC backbone between 0.82–1.15 ppm and maleimide protons (2H, peak k) at 6.8 ppm. The DP, yield, and modification ratio of each PMPC polymer were summarized in Supplementary Table S1. To produce thiol residues on the antibody, we incubated Tmab with excess TCEP (Figure 1B) and confirmed the disulfide-cleavage with SDS-PAGE since SDS can break down the van der Waals force and hydrogen bonds that hold the light chain and heavy chain together after disulfide-cleavage (Schägger, 2006). As shown in the SDS-PAGE image, Tmab was broken down into two distinct fragments with molecular weights of 50 KD and 25 KD, representing heavy and light chains, respectively (lane 2, Figure 2A). The average number of free thiol residues per Tmab following TCEP cleavage was calculated from the Ellman assay to be 7.1 per Tmab.

To prepare Tmab PMPC conjugates (Tmab^PMPC^s), we mixed the disulfide bonds-cleaved Tmab with maleimide-modified PMPC polymers (Figure 1B). The conjugates with corresponding PMPC lengths were denoted as Tmab^PMPC50^, Tmab^PMPC100^, and Tmab^PMPC200^, respectively. The PMPC conjugation shifts up bands depending on the feed ratios of the monomer MPC (lanes 3–5, Figure 2A), validating the successful PMPC conjugation. The average conjugation numbers of the polymer chains per antibody are 5.8, 4.7, and 4.2, which were determined by the average consumed thiol number after the conjugation through the Ellman assay. To compare with the MPC nanoencapsulation methodology, we also synthesized MPC nanoencapsulated Tmab (nTmab), in which we fabricated an MPC polymer network was fabricated surrounding the antibody surface via *in situ* polymerization (Wu et al., 2019). The size of each Tmab^PMPC^s was determined by DLS measurements (Figure 2B). Compared to the average diameter of native Tmab (8 nm), that of nTmab was 22 nm. Whereas the average diameters of Tmab^PMPC^s were 8–12 nm, which is the same or slightly larger compared to native Tmab. PMPC conjugation did not change the size intensely compared to MPC nanoencapsulation, implying a partial surface coverage by conjugated PMPC chains rather than a full coverage in nTmab.

We next assessed the impact of PMPC chain length on the biofunctionalities of Tmab. The mechanisms of action of Tmab in HER2+ tumor elimination mainly fall into three categories: 1) blockade of the surface HER2 receptor on tumor cells (Yarden, 2001); 2) disappearance of the HER2 receptor on the cell surface after endocytosis (Maadi et al., 2021); 3) antibody-dependent cell-mediated cytotoxicity (ADCC) and phagocytosis (ADCP) (Su et al., 2018). To investigate the HER2-binding affinity after PMPC conjugation, we fluorescently labeled Tmab^PMPC^s with AF647 and incubated them with the HER2+ human ovarian cancer cell line SKOV-3. Antibody binding was assessed by CLSM imaging and flow cytometry. CLSM images indicated that the affinity of Tmab^PMPC^s decreased as the length of PMPC increased, which was more apparent in Tmab^PMPC200^ than in the other two Tmab^PMPC^s (Figure 2C). We did not observe any binding of AFTmab on HER2-cell lines, including MDA-MB-231 or OVCAR3 cells (data not shown). The loss of binding to SKOV-3 cells was also validated by flow cytometry (Figure 2D). There was a modest change in Kd of Tmab^PMPC^s depending on the PMPC chain length; Kd of Tmab^PMPC50^, Tmab^PMPC100^, Tmab^PMPC200^ was 1.2, 1.6, and 4.9 nM, respectively, which were comparable to that of Tmab (0.9 nM). Whereas nTmab did not show HER2-dependent binding due to the strong cytophobicity (Liang et al., 2016).

We next assessed the impact of PMPC conjugation on antibody internalization (Figure 2E). AF647-labeled Tmab^PMPC^s were incubated with SKOV-3 cells for 24 h, followed by staining the Tmab on the cell surface with AF488-labeled anti-human IgG antibody. Neglectable AF488 fluorescence was observed on the cell surface incubated with Tmab, Tmab^PMPC50^, and Tmab^PMPC100^. In contrast, a definitive AF488 signal was seen in the case of Tmab^PMPC200^, which was colocalized with AF647 signals (Figure 2E). Pearson analysis revealed the highest degree of co-localization in the case of Tmab^PMPC200^, indicating that the conjugation of PMPC50 or PMPC100 did not interfere with the internalization of Tmab (Figure 2F). Lastly, we measured the capability of Tmab to induce ADCP. The assay was performed by using THP-1 cells as the phagocytic cell line and HER2+ mCherry + Jurkat cells as the target cell line. The Jurkat cells were pre-incubated with each Tmab sample (5 μg/mL), followed by the incubation with Far-Red labeled THP-1 cells. Human IgG was used as a negative control. The levels of ADCP were determined by the percentage of THP-1 with phagocytosed Jurkat cells. Tmab, Tmab^PMPC50^, and Tmab^PMPC100^ exhibited similar levels of phagocytosis activity, which were higher than those of IgG1 control, nTmab, or Tmab^PMPC200^. These results indicate that PMPC conjugation with shorter chains does not interfere with the induction of ADCP.

### PMPC conjugation enhances BBB penetration of Tmab through the transcytosis pathway

**FIGURE 3:**
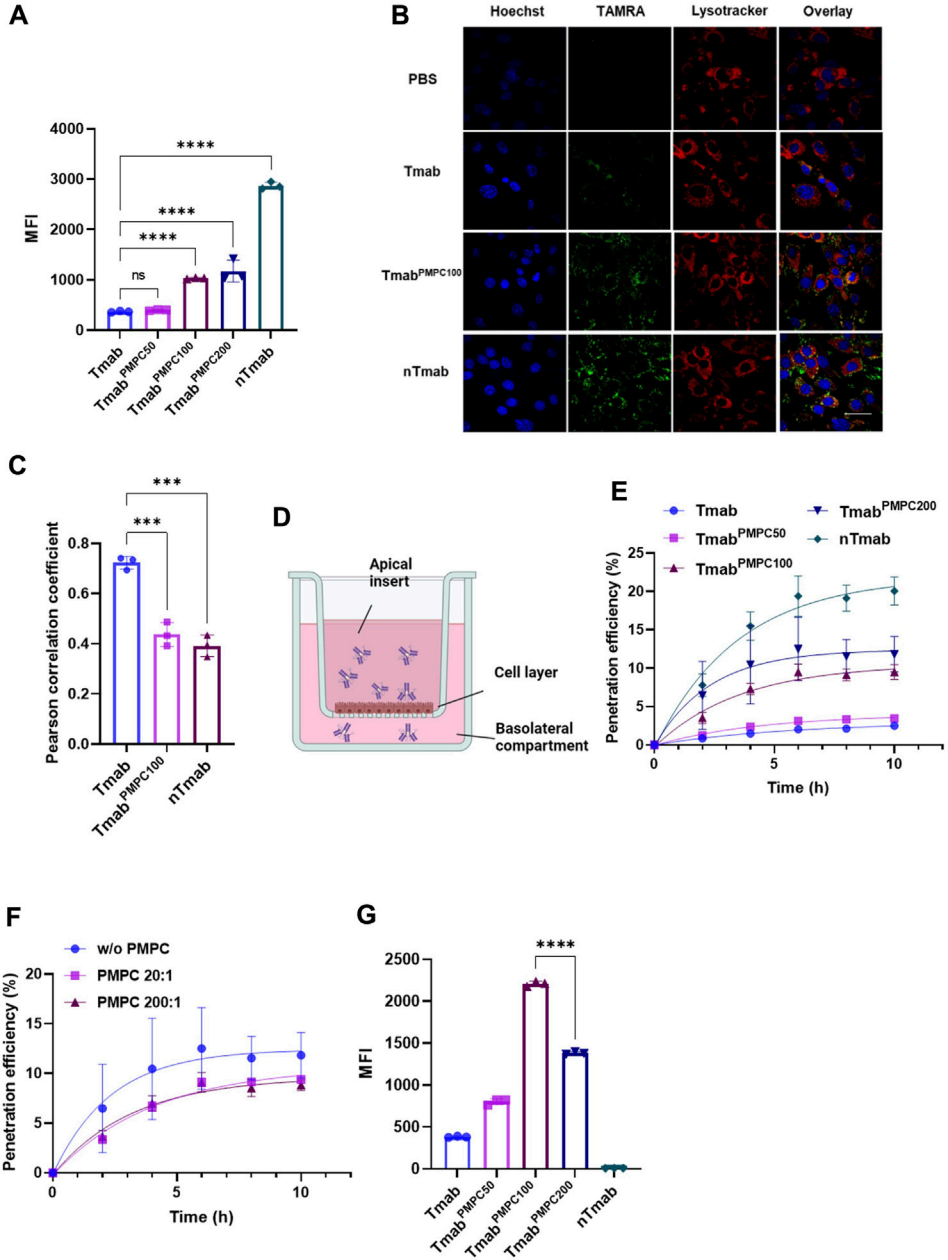
Internalization and penetration of Tmab^PMPC^ in mouse brain endothelial cells (bEnd.3). **(A)** Cellular internalization of Tmab, Tmab^PMPC50^, Tmab^PMPC100^, Tmab^PMPC200^, and nTmab in bEnd.3 cells. Each sample was labeled with TAMRA dye and incubated with bEnd.3 cells for 2 hours at 37°C. The cells were then harvested and subjected to flow cytometry to measure the MFI of TAMRA. **(B)** CLSM images showing intracellular trafficking of TAMRA (green) labeled Tmab, Tmab^PMPC100^, and nTmab in bEnd.3 cells. The cells were incubated with each sample for 4 hours at 37°C, followed by nuclear staining with Hoechst 33342 (blue) and lysosome/late endosomal staining with lysotracker deep red (red). The scale bar represents 50 μm. **(C)** Pearson correlation coefficient of lysotracker and TAMRA signals in **(B)** figures. Three different areas of each sample were analyzed. **(D)** Scheme to illustrate the experimental procedure of the transwell assay. **(E)** Accumulative penetration efficiency of TAMRA labeled Tmab, Tmab^PMPC50^, Tmab^PMPC100^, Tmab^PMPC200^, and nTmab through a monolayer of bEnd.3 cells in transwells. The fluorescence intensity in the basolateral compartment was measured at designated time points with a plate reader. **(F)** Penetration efficiency of Tmab^PMPC200^ through the bEnd.3 layers with or without PMPC200 competition. PMPC200 was mixed with Tmab^PMPC200^ at a molar ratio of 20:1 or 200:1 and added to the apical surface of the transwell. **(G)** Binding affinity of penetrated Tmab, Tmab^PMPC50^, Tmab^PMPC100^, Tmab^PMPC200^, and nTmab to SKOV-3 cells. The medium in the basolateral compartment was collected 10 hours post sample addition and incubated with SKOV-3 cells. The MFI was measured by flow cytometry. Error bars represent the s.e.m. of triplicate samples (n = 3), ****p < 0.0001.

PMPC facilitates BBB penetration through receptor-mediated transcytosis in BBB endothelial cells (Wu et al., 2019). To study the *in vitro* BBB penetration efficiency of Tmab^PMPC^, we incubated bEnd.3 cells with TAMRA-labeled Tmab^PMPC^s. Tmab and nTmab were also included as controls. The levels of internalization in bEnd.3 cells were determined by flow cytometry for TAMRA MFI. The MFI of TAMRA for Tmab^PMPC100^ or Tmab^PMPC200^ was significantly higher than for Tmab or Tmab^PMPC50^, indicating that enhanced internalization of Tmab was achieved through conjugation of longer PMPC chains (Figure 3A). To validate that the internalized Tmab^PMPC^ proceeded to the transcytosis pathway, we stained the late endosome and lysosome of bEnd.3 cells with lysotracker. More of the TAMRA signals were localized outside of the late endosome or lysosome in bEnd.3 cells treated with Tmab^PMPC100^ or nTmab. In contrast, the signals of Tmab were overlapped with the lysotracker signals in CLSM images (Figure 3B). Quantitative Pearson analysis revealed that Tmab^PMPC100^ and nTmab had significantly lower co-localization with the late endosome/lysosome compared to Tmab (Figure 3C). This suggests that the internalized Tmab^PMPC^ proceeds to the transcytosis pathway and is not trapped in endosomal compartments. We next quantified levels of transcytosis using bEnd.3 transwell assay as reported (Wu et al., 2019) (Figure 3D). Tmab samples labeled with TAMRA were added to the apical inserts of the transwell. The medium in the basolateral chamber was collected at designated time points, and the TAMRA fluorescent intensity was measured (Figure 3E). The penetration of Tmab^PMPC100^ and Tmab^PMPC200^ reached approximately 10% after 10 h incubation, which was lower than that of nTmab (20%) but remarkably higher than those of Tmab and Tmab^PMPC50^ (2% and 2.3%). Transcytosis of Tmab^PMPC^ was strongly interfered in the presence of the free PMPC200, suggesting that the binding of PMPC to bEnd.3 cell layers was required for this step (Figure 3F). These findings collectively proved that the conjugation of PMPC to Tmab enhanced BBB penetration through PMPC-mediated transcytosis *in vitro*. To confirm that Tmab maintains the epitope affinity following the transcytosis, we incubated SKOV-3 cells with the medium in the basolateral chamber 10 h post sample addition to the apical chamber and measured the MFI by flow cytometry (Figure 3G). Both Tmab^PMPC100^ and Tmab^PMPC200^ showed a higher MFI than that of Tmab or nTmab, indicating that the HER2 binding of Tmab was maintained after transcytosis. Although the penetration efficiency of Tmab^PMPC200^ is slightly higher than that of Tmab^PMPC100^ in terms of absolute antibody amount in the basolateral chamber (see Figure 3E), the penetrated Tmab^PMPC100^ exhibits the highest MFI in SKOV-3 cells due to its better retainment of the epitope recognition compared to that of Tmab^PMPC200^. This indicates that PMPC100 is a superior to achieve efficient BBB penetration while retaining the epitope recognition.

### PMPC conjugation enhances the brain delivery of Tmab

**FIGURE 4:**
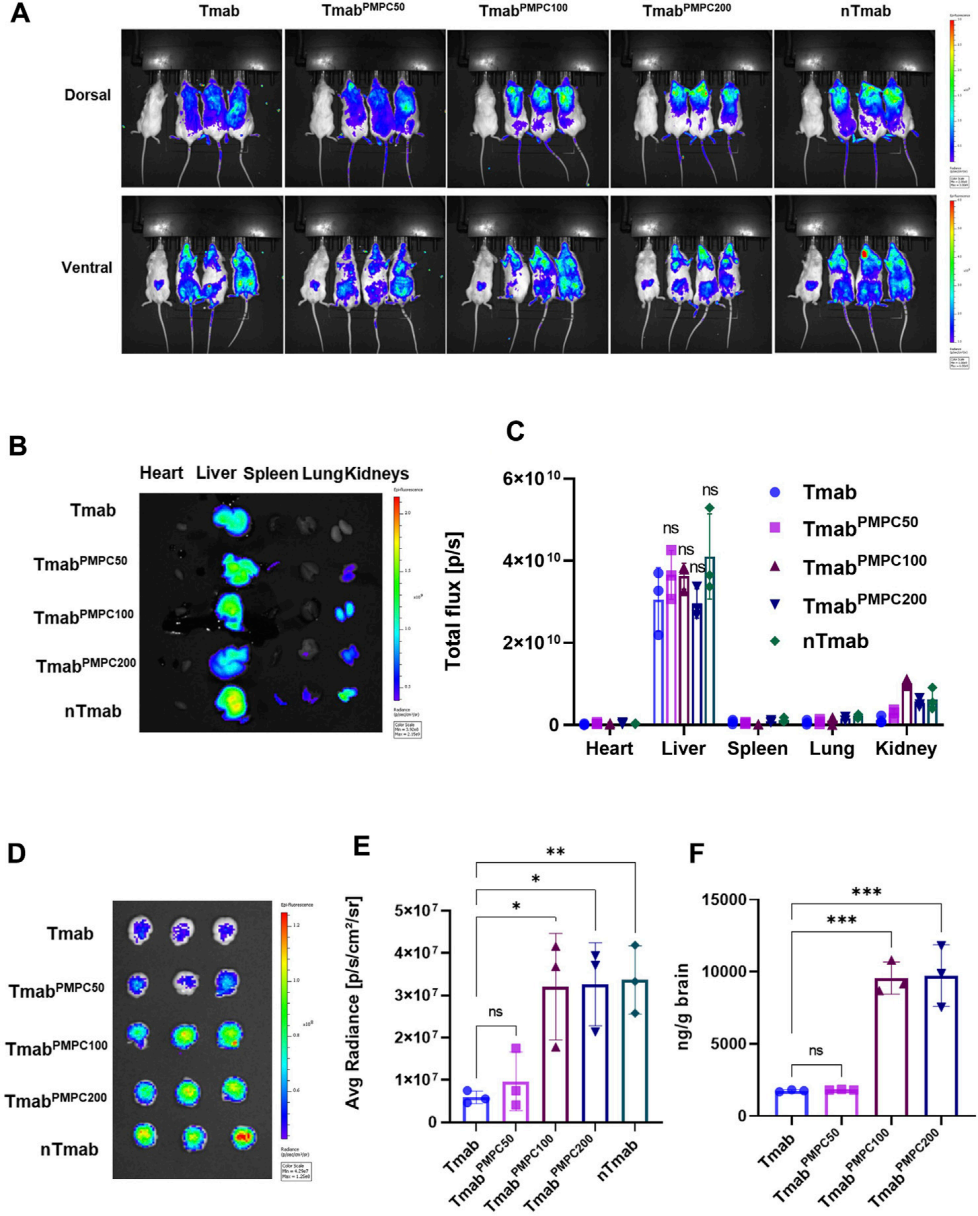
Biodistribution and brain delivery of Tmab^PMPC^. NSG mice were treated with AF647-labeled Tmab, Tmab^PMPC50^, Tmab^PMPC100^, Tmab^PMPC200^, nTmab, and PBS (100 μL) via retro-orbital injection (10 mg/kg). **(A)** Whole body imaging of AF647 fluorescence by IVIS imaging. The mouse on the far-left side of each image was treated by PBS. The mice were perfused with PBS as in the Materials and Methods, and the organs (liver, lung, kidneys, spleen, heart, and brain) were harvested 24 h post-injection for organ imaging and subsequent ROI measurement in B-D. **(B)** AF647 visualization by IVIS imaging, including the heart, liver, spleen, lung, and kidneys from the mice shown in **(A)**. **(C)** Total flux (photons/s) of the dissected organs. **(D)** IVIS images of the brains dissected from the mice. The mice were perfused with PBS, and the brains were harvested 24 h post-injection. **(E)** Total flux (photons/s) of the dissected brains. **(F)** Tmab concentrations in the brains following PBS perfusion. The brains were weighed and homogenized after IVIS imaging. The amount of Tmab in the brains was quantified by sandwich ELSA with anti-human IgG antibodies. Error bars represent the s.e.m. of triplicate samples (n = 3), ****p < 0.0001.

We next assessed the delivery of Tmab^PMPC^s to the brain *in vivo* using NSG mice. The signals from free AF647 dye were negligible in comparison to that from AFTmab in mice via the retro-orbital injection, indicating that AF647 dye did not make a false signal in this model (Supplementary Figure S3). AF647-labeled Tmab^PMPC^s, Tmab, or nTmab were administered to mice via the retro-orbital vein, and the fluorescent signals were imaged by IVIS imaging (Figure 4A). Native Tmab, as well as Tmab^PMPC50^ were evenly distributed in mice. In contrast, Tmab^PMPC100^ and Tmab^PMPC200^ were enriched around the head area similar to nTmab 24 h post-injection. To confirm the organ distribution of each form of Tmab, we harvested the heart, liver, spleen, lung, and kidneys from these mice following PBS perfusion as in the Materials and Methods. The organ images (Figure 4B) and the fluorescence of ROI in total flux (p/s) (Figure 4C) indicated that the liver was the primary organ for Tmab accumulation, while PMPC conjugation induced no significant change in the level of liver accumulation (Figure 4C, ns). These results indicated the PMPC conjugation did not change the non-specific clearance of Tmab by the reticuloendothelial system (RES) in the liver. We also analyzed the brains of these mice and measured AF647 intensity by IVIS imaging. The brain images (Figure 4D) and average fluorescent radiance (p/s/cm^2^/sr) (Figure 4E) indicated that Tmab^PMPC100^, Tmab^PMPC200^, and nTmab had a similar average radiance that were over 5.5-fold higher than that of Tmab, indicating that PMPC conjugation with longer chain lengths significantly enhanced brain penetration of Tmab (Note: the fluorescent intensity measurement from IVIS images is a semi-quantitative method due to the limited penetration depth of fluorescence in tissues). To further quantify the deposited Tmab level in the brain, we homogenized brain tissues after imaging and titrated the Tmab concentration by sandwich ELISA assay with anti-human IgG1 antibodies. Brain extracts from PBS-treated mice were used to determine the background signal intensity, which was subtracted from the results of those Tmab samples. Samples obtained from mice treated with Tmab^PMPC100^ and Tmab^PMPC200^ contained over 4-fold higher Tmab than that of native Tmab or Tmab^PMPC50^, which was consistent with the quantitative ROI data (Figure 4F). Those data collectively indicated that Tmab^PMPC^s with longer chain lengths enhanced brain delivery of Tmab, which was comparable to that of the nanocapsule.

### PMPC conjugation does not induce neurotoxicity

**FIGURE 5:**
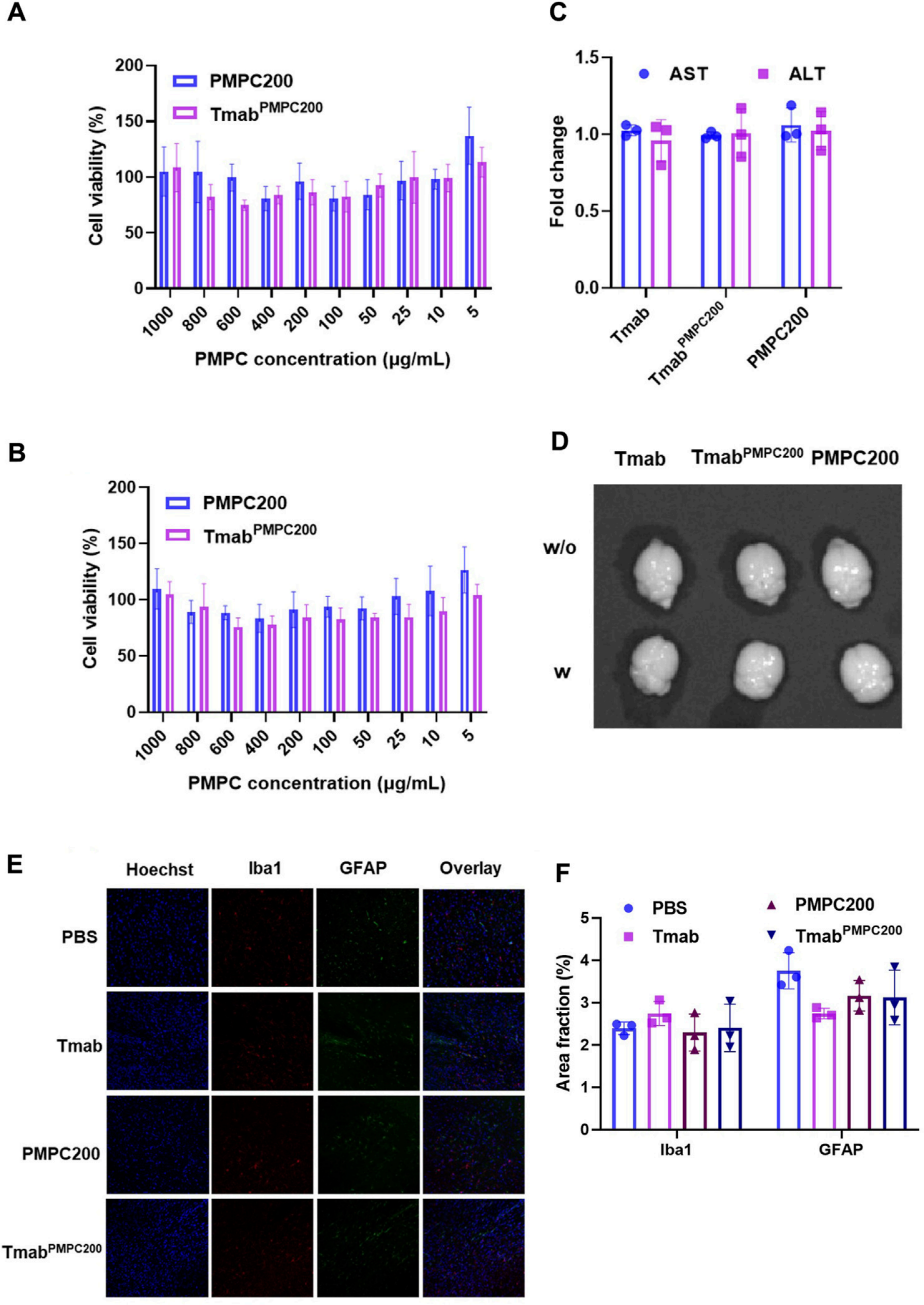
*In vitro* and *in vivo* toxicity assays of Tmab^PMPC^. *In vitro* cytotoxicity assay on **(A)** bEnd.3 and **(B)** JX14P cells. Cells were incubated with PMPC200 or Tmab^PMPC200^ at the PMPC concentrations ranging from 5 to 1,000 μg/mL for 72 h. The cellular viability was measured by CCK-8 assay. **(C)** Fold changes of AST and ALT in the plasma of the mice treated with Tmab, PMPC200, or Tmab^PMPC200^. The Tmab dosage was 50 mg/kg while the PMPC dosage was 90 mg/kg, and the plasma was collected before and 72 h post-treatment. **(D)** Mice were treated with Tmab, PMPC200, and Tmab^PMPC200^. Evans blue dye solution (4 mg per mouse) was intravenously injected to mice 24 h post-treatment, and the brains were obtained 2 h post-Evans blue dye injection. The brain images were taken by IVIS imaging. **(E)** Immunofluorescent images of the brain sections from the mice treated with Tmab, PMPC200, or Tmab^PMPC200^. The brains were collected 72 h post-treatment and processed to sections with a vibratome, followed by staining with anti-Iba1 and anti-GFAP primary antibodies and the corresponding dye-labeled secondary antibodies. The nuclei were visualized by Hoechst 33342 staining. **(F)** Quantification of the Iba1 or GFAP positive area fractions from the images in **(E)**.

We next assessed the toxicities of Tmab, PMPC polymer (PMPC200), and Tmab PMPC conjugate (Tmab^PMPC200^) *in vitro* and *in vivo* assays. The *in vitro* cytotoxicity assay was performed using two different cell lines; bEnd.3 cells as the BBB endothelial cell model cell and JX14P cells derived from a glioblastoma patient brain biopsy as the glial cell model. Cells were incubated with Tmab, PMPC200, or Tmab^PMPC200^ for 72 h. The cytotoxicity was determined by cellular viability assay with a CCK-8 assay kit (Figures 5A,B). Neither PMPC200 nor Tmab^PMPC200^ exhibited detectable toxicity on both cells at the PMPC concentrations ranging from 5 to 1,000 μg/mL. The toxicity was also evaluated *in vivo* using NSG mice. Mice were injected with Tmab, PMPC200, or Tmab^PMPC200^ at the Tmab dosage of 50 mg/kg (corresponding to a dosage 4 mg/kg in humans), or PMPC dosage of 90 mg/kg (estimated by the calculation described in the Materials and Methods) or 72 h post-injection. The plasma levels of two liver enzymes, AST and ALT, were measured to evaluate liver toxicity. There was no change in the level of AST or ALT 72 h after the treatment in all three groups compared with the plasma prior to the treatment (baseline), suggesting that PMPC or its conjugate did not induce liver toxicity (Figure 5C). To rule out the possibility of BBB disruption by PMPC, we injected Evans blue dye intravenously 24 h post-treatment, which can deposit in the brain only upon disruption of the BBB. There was no sign of Evans-blue dye in the brain of mice treated with Tmab, PMPC200, or Tmab^PMPC200^, indicating that the enhanced brain delivery of Tmab via PMPC conjugation is not a result of BBB disruption (Figure 5D). At last, we tested whether PMPC conjugation induces neurotoxicity by measuring the expression of Iba1 and GFAP, as reported previously (Wen et al., 2019; Wu et al., 2019). Iba1 and GFAP were used as biomarkers for microglia and astrocytes in the brain, respectively, whose expressions increase upon brain damage (O’Callaghan and Sriram, 2005). The brains were obtained from mice treated with PBS (control), Tmab, PMPC200, or Tmab^PMPC200^ at the PMPC dosage of 90 mg/kg or Tmab dosage of 50 mg/kg 72 h post-treatment and processed for immunofluorescent imaging of Iba1 and GFAP. No significant difference in levels of either Iba1 or GFAP signal density was seen in the immunofluorescent figures of the brains (Figure 5E) treated by Tmab, PMPC200, or Tmab^PMPC200^. The expression levels of Iba1 and GFAP quantified by area fraction analysis also supported this observation (Figure 5F). Those findings collectively indicated that PMPC conjugation achieved effective brain delivery of Tmab without induction of adverse effects, at least in the liver, the BBB, or the brain.

## Discussion

Although antibodies have emerged as the major player in precision medicine, their applications are highly limited in treating brain diseases due to the presence of the BBB. We have developed a novel nanodelivery platform for antibody drugs using polymer technologies. In the previous study, antibody surface was concealed by the PMPC network, thus necessitating additional antigen-targeting ligands and degradable crosslinkers to re-direct the nanocapsule to the targeting site and restore the antibody functions in the brain. The complexity of this system brings about difficulties in medical translations. We here successfully developed a simple methodology that enabling comparable brain delivery of antibody as the previous nanodelivery platform via direct conjugation of the brain-penetrable polymer, PMPC, to specific sites of Tmab, which can address most of the issues of the previous platform listed above. PMPC100 allowed effective delivery of Tmab to the brain via enhanced BBB penetration while preserving its essential functionalities, such as epitope recognition, receptor-mediated internalization, and effector function. Moreover, we did not observe detectable levels of toxicities in cell culture and mice treated with Tmab-conjugated with PMPC, indicating that this simple methodology for PMPC engineering of human IgG1 confers a brain-penetrable moiety more safely and should be highly beneficial for future clinical translation. Such a methodology readily converts antibody therapeutics to a brain-deliverable form while maintaining their medical functionalities in the brain. The concerns of brain-entry issue haunt the development of brain-disease-targeting antibody therapeutics, impeding the medical translations of laboratory-generated antibodies to clinical practices. In this context, this simple methodology has great potential to serve as the platform, to not only repurpose the current antibody therapeutics but also encourage the design of novel antibodies for the treatment of brain diseases.

Low-RES clearance is the prerequisite for targeting reagents to guarantee their targeting efficiency, which is one of the advantages of antibodies over other types of targeting reagents. A group of BBB-penetrating strategies has been reported to enhance brain delivery of therapeutic antibodies. However, many of them are peptide-based ligands that are either hydrophobic or highly charged. Such modifications induce non-specific accumulation and disrupt their long-circulating profiles, resulting in poor delivery to the target sites. PMPC is a super-hydrophilic polymer that is neutrally charged at physiological pH values. The conjugation does not change the biodistribution of Tmab in the liver, indicating that PMPC modification does not induce the RES clearance of the antibody and, thereby, could achieve a higher antigen-specific targeting efficiency than other brain targeting ligands.

A typical IgG1 antibody contains two identical light and heavy chains. It includes 16 disulfide bonds, including four interchain disulfide bonds in the hinge region and 12 intrachain disulfide bonds associated with 12 individual domains (Liu and May 2012). Among those disulfide bonds, the interchain bonds are preferably cleaved by TCEP, providing eight free thiol residues. The results from Ellman assays to determine the thiol number indicated that an average of 7.1 thiol residues were produced from one Tmab molecule while four to five residues were consumed by PMPC conjugation. Tmab functionality results indicated that the thiol-residue-specific PMPC conjugation minimized the function loss of the antibody compared with MPC nanocapsulation. Among the PMPC polymers with different degrees of polymerization, Tmab^PMPC50,^ and Tmab^PMPC100^ exhibited almost comparable antibody functions as Tmab, suggesting that PMPC conjugation with an optimal length can retain the antibody functions. These studies would genuinely allow site-specific PMPC conjugation on therapeutic antibodies, which will be beneficial for further tuning of PMPC modification.

The MPC polymer network enhanced brain delivery of therapeutic antibodies through receptor-mediated transcytosis. The surface ligand density was the most dominant variable determining the binding strength. One concern is that the conjugated PMPC, with a linear polymer topology and partial coverage of the antibody surface, could have a lower MPC density and be insufficient to enhance brain delivery effectively. The results from both *in vitro* BBB penetration using bEnd.3 cell layer and *in vivo* brain delivery experiments showed that Tmab^PMPC100^ and Tmab^PMPC200^ exhibited comparable levels of BBB penetration and brain deposition of Tmab, which were identical to those of nTmab. These results indicated that the MPC density in Tmab^PMPC100^ was comparable to that in nTmab, which was required to achieve effective brain delivery. Due to the retainment of epitope recognition following transcytosis, PMPC100 is the optimal chain length to enable enhanced antibody brain delivery with preserved functionality.

It should be noted that TCEP did not selectively cleave the disulfide bonds in the hinge area or those between the light chain and heavy chain. In the future, a more selective disulfide-cleavage methodology (e.g., reduction by TCEP immobilized agarose beads) will be employed to cleave the disulfide bonds between the light chain and heavy chain for a more specific PMPC conjugation. In addition, the conjugation sites and the polymer length are optimized in Tmab, which is the IgG1 subtype. However, the number and locations of interchain disulfide bridges differ in other IgG subtypes (Liu and May 2012), which may lead to variations in antibody function and brain delivery efficiency of non-IgG1 antibody PMPC conjugates. Further optimization would be necessary to apply this methodology to non-IgG1 antibodies. In conclusion, we developed a novel but a simple delivery methodology for therapeutic antibodies of the IgG1 subtype, enabling their effective delivery to the brain while maintaining various functionalities. This affordable methodology could be extended to other therapeutic antibodies beyond Tmab and benefit the treatment of various brain diseases.

## Data Availability

The original contributions presented in the study are included in the article/Supplementary Material, further inquiries can be directed to the corresponding author.
