# Supplementary material for: Site-oriented conjugation of poly(2-methacryloyloxyethyl phosphorylcholine) for enhanced brain delivery of antibody
Source: Front Cell Dev Biol. 2023 Oct 18;11:1214118. doi: 10.3389/fcell.2023.1214118 (PMC10618420; doi:10.3389/fcell.2023.1214118)
Supplement: Supplementary file 1 [file DataSheet1.PDF]

Supplemental figures

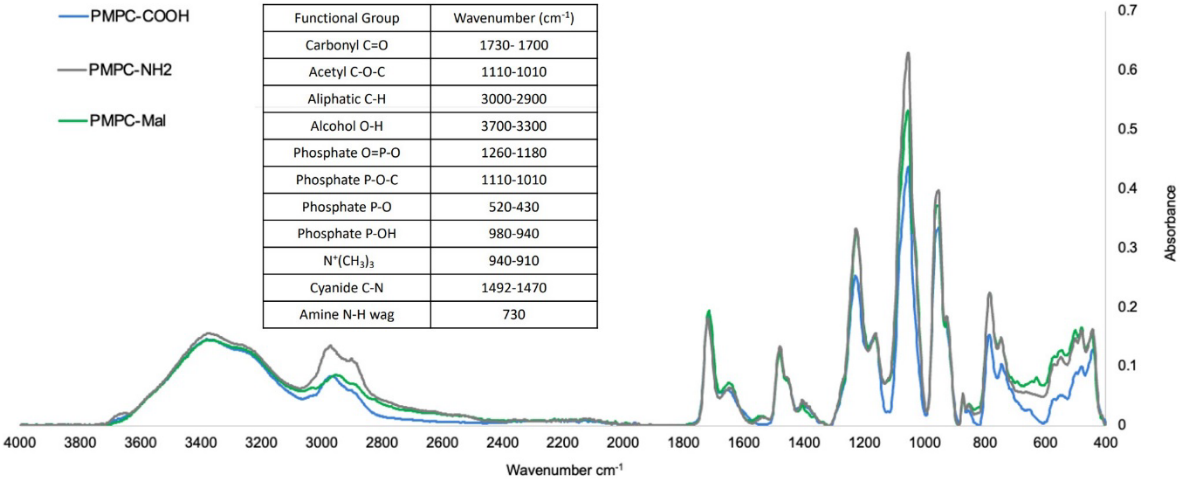

Functional Group	Wavenumber (cm^{-1})
Carbonyl C=O	1730- 1700
Acetyl C-O-C	1110-1010
Aliphatic C-H	3000-2900
Alcohol O-H	3700-3300
Phosphate O=P-O	1260-1180
Phosphate P-O-C	1110-1010
Phosphate P-O	520-430
Phosphate P-OH	980-940
$N^{+}(CH_3)_3$	940-910
Cyanide C-N	1492-1470
Amine N-H wag	730

Supplemental Figure 1. FT-IR spectra of PMPC-COOH, deprotected PMPC, and PMPC-Mal.

The functional chemical species presenting in PMPC polymer were characterized by FT-IR analysis using a Bruker Alpha ATR-FTIR spectrometer. The ATR accessory was used for signal enhancement of chemical species by internal total reflectance. The number of scans was set to 32 with a resolution on four for all samples analyzed.

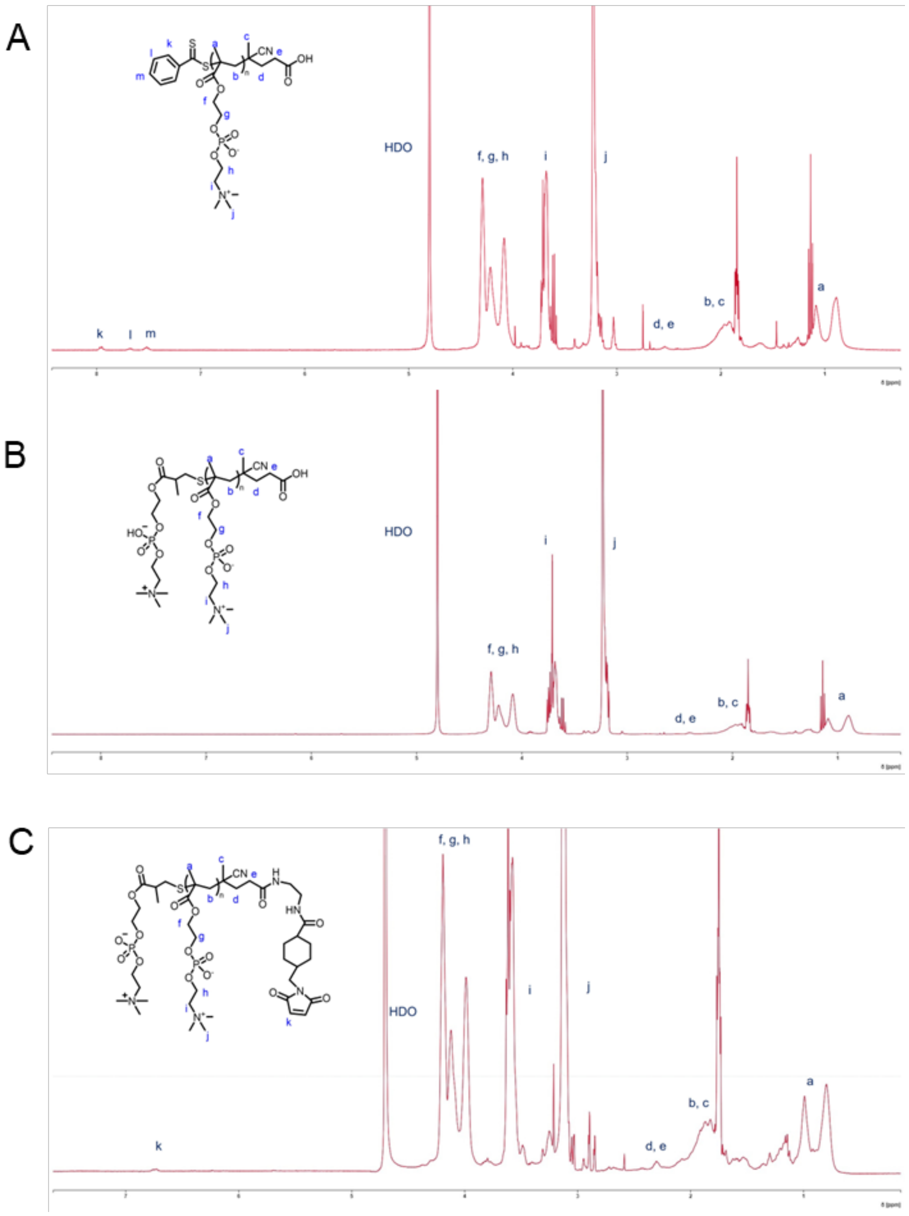

Supplemental Figure 2. The ^{1}H NMR spectra of a) PMPC100-COOH, b) deprotected PMPC100-COOH, and c) PMPC100-Mal.

The number of repeat units in the PMPC backbone was characterized by proton nuclear magnetic resonance spectroscopy (^{1}H NMR) analysis using a Bruker 400 MHz NMR spectrometer. The PMPCs were prepared in D_2O at a concentration of 1 mg/mL and analyzed at room temperature with 64 scans for determination of degree of polymerization.

Additionally, the percent conversion from PMPC-COOH to PMPC-Mal was calculated by ^{1}H-NMR analysis, and the PMPC-Mal samples were prepared for ^{1}H-NMR and with 256 number of scans to resolve the end group proton signals appearing at δ = 6.8 ppm.

PMPC100-COOH: ^{1}H NMR (400 MHz, D_2O, δ) from one side: 0.7-1.1 (a, 3H, -C*CH3*), 1.6-2.2 (b, 2H, -*CH2*C-; c, 3H, -C*CH3*), 2.4-2.8 (d and e, 4H, HOOC*CH2CH2*-), 3.2 (j, 9H, -N*(CH3)3*), 3.6 (i, 2H, -*CH2*N-), 3.8-4.2 (f, g, and h, 6H, -COO*CH2CH2*OPO*CH2*-), 7.4-8.0 (k, l, and m, 5H, -C*CHCHCHCHCH*).

Deprotected PMPC100-COOH: ^{1}H NMR (400 MHz, D_2O, δ) from one side: 0.7-1.1 (a, 3H, -C*CH3*), 1.6-2.2 (b, 2H, -*CH2*C-; c, 3H, -C*CH3*), 2.4-2.8 (d and e, 4H, HOOC*CH2CH2*-), 3.2 (j, 9H, -N*(CH3)3*), 3.6 (i, 2H, -*CH2*N-), 3.8-4.2 (f, g, and h, 6H, -COO*CH2CH2*OPO*CH2*-).

PMPC100-Mal: ^{1}H NMR (400 MHz, D_2O, δ) from one side: 0.7-1.1 (a, 3H, -C*CH3*), 1.6-2.2 (b, 2H, -*CH2*C-; c, 3H, -C*CH3*), 2.4-2.8 (d and e, 4H, HOOC*CH2CH2*-), 3.2 (j, 9H, -N*(CH3)3*), 3.6 (i, 2H, -*CH2*N-), 3.8-4.2 (f, g, and h, 6H, -COO*CH2CH2*OPO*CH2*-), 6.8 (k, 2H, -C*CHCH*C-).

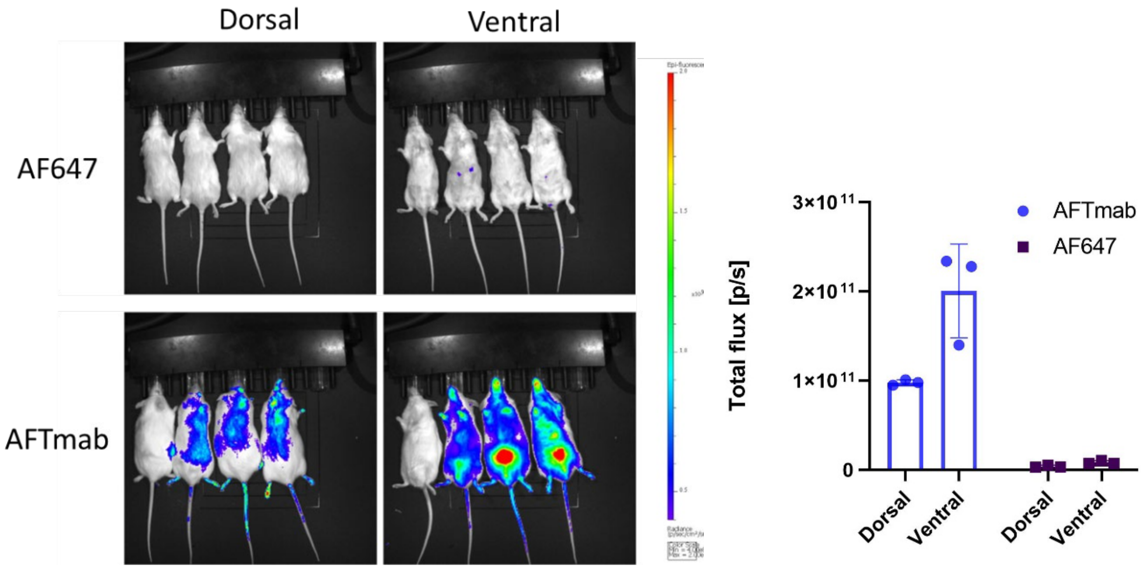

Supplemental Figure 3. Whole body imaging of NSG mice treated with AF647 or AFTmab.

Mice (n=3) were retro-orbitally injected with 100 μL AFTmab (10 mg/kg) or AF647 in PBS. AF647 was used as equivalent to the total fluorescent intensity of AFTmab. The mouse on the far left was treated with PBS as a negative control (n=1). The images were taken on an IVIS imaging system 12 hours post-injection.

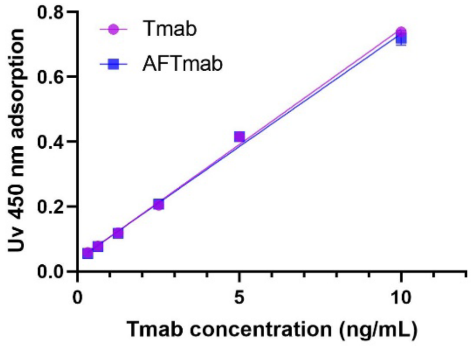

Supplemental Figure 4. ELISA standard curves of Tmab and AFTmab.

A 96 ELISA well-plate was coated with anti-human IgG antibody (1 µg/mL in carbonate buffer, pH 9.5) at 4 ^{o}C overnight, followed by three washes with PBST (0.1% Tween in PBS). Non-specific binding was blocked with blocking buffer (2% BSA in PBST) at room temperature for two hours, followed by three washes with PBST. Tmab and AFTmab were diluted with blocking buffer (concentration ranging from 0 to 10 ng/mL) and incubated in the well at room temperature for two hours, followed by five washes with PBST. HRP-coupled anti-human IgG antibody (10 µg/mL) was added to each well and incubated at room temperature for one hour, followed by five washes with PBST. Finally, 3,3',5,5'-Tetramethylbenzidine and H_2O_2 solutions were added and incubated at room temperature for 15 minutes. The UV adsorption at 450 nm was measured with a Varioskan LUX plate reader.

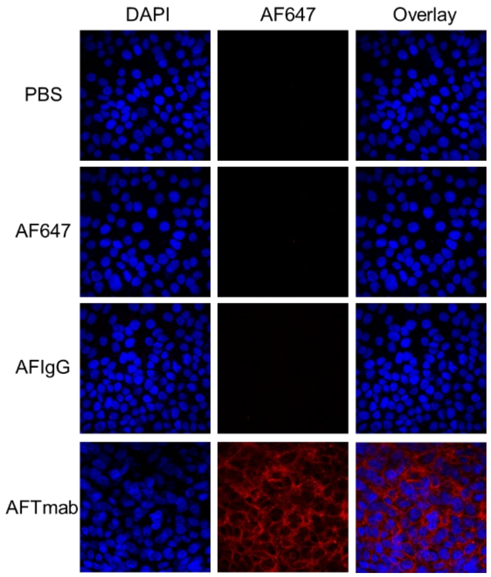

Supplemental Figure 5. CLSM images of SKOV-3 cells treated with PBS, AF647, AFIgG, or AFTmab.

SKOV-3 cells (2 x 10^5) were seeded on glass coverslips in 12-well plate. After 24 hours, cells were incubated with AFTmab (2 μg/mL), AF647, AFIgG (equivalent to the fluorescent intensity of AFTmab), and PBS (10 μL) at 37 ^{o}C for two hours, followed by 4% paraformaldehyde for 15 minutes at room temperature for fixation after three washes with PBS. Nuclei were stained by 10 μg/mL Hoechst 33342 at room temperature for 20 minutes. Fluorescent images were taken with a Nikon A1R/SIM confocal microscope.

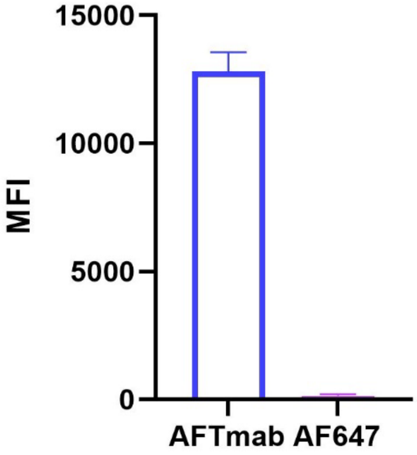

Supplemental Figure 6. Tmab-dependent binding of AFTmab to SKOV-3 cells.

SKOV-3 cells (1 x 10^5) were incubated with AFTmab (2 μg/mL) and AF647 (equivalent to the fluorescent intensity of AFTmab) for two hours at 37 °C. The cells were then washed by PBS, harvested, and fixed. The mean-fluorescent intensity of the cell surface was measured by flow cytometry.

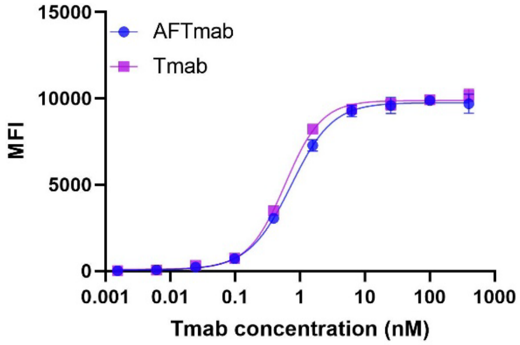

Supplemental Figure 7. Assay for Tmab-saturation binding.

SKOV-3 cells (2 x 10^5) in FACS buffer were included with AFTmab and Tmab (concentration ranging from 0.001 to 300 nM) for two hours at 4 ^oC, followed by three washes with PBS. The mean-fluorescent intensity of the cell surface was measured by flow cytometry.

Entry	MPC: CTA: VA-044 (molar ratio)	Conversion ratio (%)	modification ratio (%)	Yield (%)
PMPC50-COOH	50: 1: 0.2	74	--	62
PMPC100-COOH	100: 1: 0.2	56	--	48
PMPC200-COOH	200: 1: 0.2	78	--	74
PMPC50-Mal	--	--	60.9	51
PMPC100-Mal	--	--	51.9	62
PMPC200-Mal	--	--	11.3	79

Supplemental Table 1. The summary of DP, yield, and modification ratio of PMPC-COOHs, and PMPC-Mals with different designated feed ratios.
